# Fine-Tuning the Processes of Steroid Hormone Biosynthesis: SULT2A1/1E1 and CYP21A2 Interaction

**DOI:** 10.3390/ijms27219754

**Published:** 2026-10-30

**Authors:** Evgeniy Yablokov, Anastasiya Tumilovich, Oksana Gnedenko, Diana Mednaia, Yuri Mezentsev, Pavel Ershov, Mihail Konstantinov, Leonid Kaluzhskiy, Tatsiana Tsybruk, Irina Grabovec, Polina Shabunya, Andrei Gilep, Alexis Ivanov

**Affiliations:** 1 Institute of Biomedical Chemistry, 10 Building 8, Pogodinskaya Street, 119121 Moscow, Russia; gnedenko.oksana@gmail.com (O.G.); mednayadiana@yandex.ru (D.M.); yu.mezentsev@gmail.com (Y.M.); pavel79@inbox.ru (P.E.); mishanyamihail@ya.ru (M.K.); leonid.kaluzhskiy@ibmc.msk.ru (L.K.); agilep@yahoo.com (A.G.); alexei.ivanov@ibmc.msk.ru (A.I.); 2 Institute of Bioorganic Chemistry NASB, 5 Building 2, V.F. Kuprevich Street, 220084 Minsk, Belarus; tumilovicham@gmail.com (A.T.); tvshkel@gmail.com (T.T.); graboveci71@gmail.com (I.G.); polinashabunya@gmail.com (P.S.)

**Keywords:** CYP21A2, SULT2A1, SULT1E1, steroid hormone biosynthesis, protein–protein interactions, surface plasmon resonance, enzymatic activity modulation, molecular modeling

## Abstract

The high physiological activity of steroid hormones requires multilevel regulation of their biosynthesis. Cytosolic sulfotransferases, specifically SULT2A1 and SULT1E1, convert steroid precursors into mobile sulfated depot forms, diverting them from key steroidogenic cytochromes P450. Given the previously demonstrated CYP17A1–SULT interactions, this study aims to verify the hypothesis that CYP21A2 can interact with SULTs and thereby potentially contribute to the regulation of steroid metabolism. Using surface plasmon resonance (SPR) biosensing, in vitro enzymatic activity assays, and in silico molecular modeling, we investigated the interplay between CYP21A2 and SULT2A1/1E1. SPR analysis revealed high-affinity binding between purified recombinant CYP21A2 and SULT2A1/SULT1E1 in vitro in the nanomolar range (9.5 × 10^−9^ M for SULT2A1 and 1.15 × 10^−8^ M for SULT1E1). Thermodynamic assessments indicated that the CYP21A2–SULT1E1 complex is entropy-driven, contrasting with the enthalpy-driven CYP17A1–SULT1E1 interaction. Enzymatic assays demonstrated reciprocal modulatory effects: CYP21A2 exerted a stimulatory effect on pregnenolone sulfation by SULTs, whereas SULT2A1 moderately inhibited CYP21A2 activity. Molecular modeling of binary complexes predicted structural interfaces and continuous intraprotein channels connecting the enzymes’ active sites. These findings suggest that CYP–SULT interactions may contribute to the fine-tuning of steroid hormone biosynthesis. These findings raise the possibility that CYP–SULT interactions may form part of a broader regulatory network, acting as metabolic switches that dictate the balance between downstream corticosteroid pathways and structural sequestration into sulfated pools.

## 1. Introduction

Steroid hormones are a group of physiologically active lipophilic substances based on a sterane scaffold and regulate key life processes in vertebrates. In the human body, there are two main groups of steroid hormones: corticosteroids (mineralocorticoids and glucocorticoids) and sex steroids. Mineralocorticoids regulate water and salt metabolism, while glucocorticoids perform multimodal regulatory functions, including modulating carbohydrate metabolism, certain immune processes, cognitive functions, and embryonic development. Sex steroids play a role in regulating the development and functioning of the reproductive system and are divided into androgens, estrogens, and progestogens.

**Figure 1:**
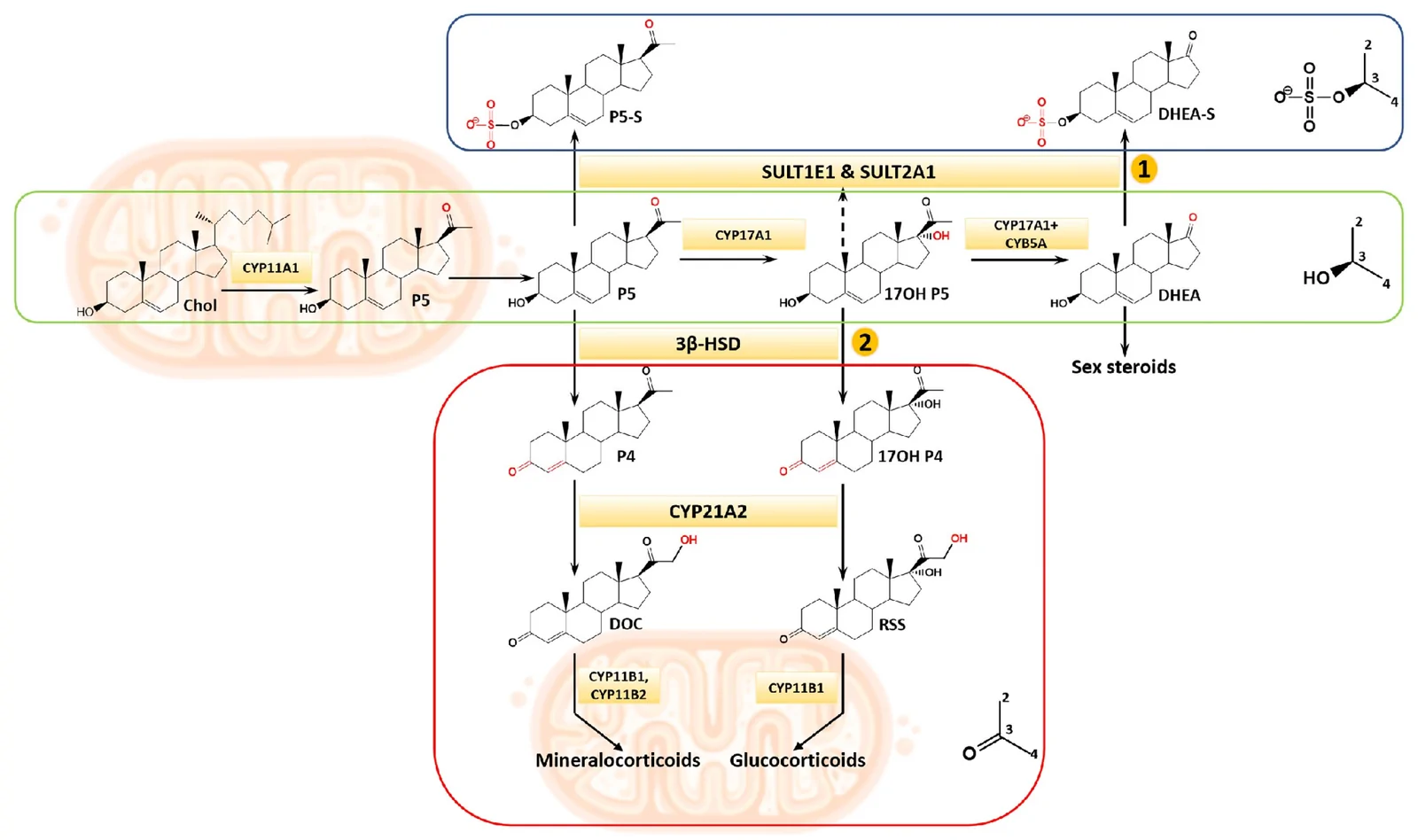
Biosynthetic pathways of major human steroid hormones highlighting C3-functional group partitioning and cytosolic sulfotransferase-mediated metabolic switches. Colored borders delineate distinct steroid groups categorized by the specific structural state of the functional group at the C3 atom of ring A within the sterol core (schematically represented on the right): the green border—3β-hydroxyl (-OH) moiety; the blue border—sulfate conjugates possessing a 3β-sulfate (-O-SO3^−^) group; and the red border—steroids featuring a 3-keto (=O) conformation. Numbered orange circles designate critical regulatory checkpoints or metabolic “switches” controlling pathway flux: (1) Switch 1 (SULT1E1 and SULT2A1 checkpoint) manages the reversible phase II sulfation of Δ^5^-intermediates, dictating the balance between immediate steroid utilization and structural sequestration into stable mobile sulfated pools (P5-S, DHEA-S); (2) Switch 2 (3β-HSD checkpoint) governs the critical commitment step wherein Δ^5^-hydroxysteroids undergo oxidation and isomerization into Δ^4^-ketosteroids, effectively gating the entry of precursors into downstream corticosteroid pathways. Key mitochondrial, microsomal and cytosolic enzymatic systems driving individual catalytic conversions are labeled along the respective metabolic transitions: CYP11A1 (scc, cholesterol side-chain cleavage enzyme), SULT1E1 (estrogen sulfotransferase), SULT2A1 (dehydroepiandrosterone sulfotransferase), CYP17A1 (steroid 17α-hydroxylase/17,20-lyase), CYB5A (cytochrome b5 type A), 3β-HSD (3β-hydroxysteroid dehydrogenase/Δ^5^–Δ^4^ isomerase), CYP21A2 (steroid 21-hydroxylase), CYP11B1 (steroid 11β-hydroxylase), CYP11B2 (aldosterone synthase). Steroid abbreviations are defined as follows: Chol (cholesterol), P5 (pregnenolone), P5-S (pregnenolone sulfate), 17OH P5 (17α-hydroxypregnenolone), DHEA (dehydroepiandrosterone), DHEA-S (dehydroepiandrosterone sulfate), P4 (progesterone), 17OH P4 (17α-hydroxyprogesterone), DOC (11-deoxycorticosterone), RSS (11-deoxycortisol, Reichstein’s Substance S).

All steroid hormones in the human body are synthesized from a common precursor, cholesterol, through the involvement of multiple enzymatic systems. Given the high physiological activity of steroid hormones and their shared precursor, the processes of steroid hormone biosynthesis require multilevel regulation. In humans, three key enzymes of steroid hormone biosynthesis can be distinguished: steroid 17α-hydroxylase/17,20-lyase (CYP17A1), steroid 21-hydroxylase (CYP21A2), and 3β-hydroxysteroid dehydrogenase/Δ^5^–Δ^4^ isomerase (3β-HSD). Many CYP17A1 substrates possess a 3β-hydroxyl group, but CYP17A1 also metabolizes steroids bearing a 3-keto group at C3. CYP21A2 hydroxylates steroids containing a keto group at the C3 position, whereas 3β-HSD converts the hydroxyl group at C3 into a keto group (Figure 1). Together with the cellular and tissue compartmentalization of these key enzymes, this substrate specificity represents one of the regulatory mechanisms governing steroid hormone biosynthesis. To carry out catalytic functions, CYP needs to interact with redox partners such as NADPH–cytochrome P450 reductase (CPR, POR, CYPOR in microsomal monooxygenase systems [1]) and adrenodoxin (Adx, FDX1 in mitochondria [2,3]). In addition, there are additional proteins that play regulatory (microsomal cytochrome b5, CYB5A) [4] or electron-transport roles (reductases of Adx or CYB5) [5,6].

In addition to enzyme specificity and cellular compartmentalization, steroid hormone biosynthesis may also be regulated by changes in the availability of steroid intermediates for individual enzymes. However, very little attention has been paid to sulfated steroids in the context of the regulation of steroid hormone and other bioactive steroid biosynthesis. It is known that two cytosolic sulfotransferases (SULTs), namely SULT2A1 and SULT1E1, are capable of sulfating some steroids (in particular, pregnenolone (P5) and DHEA [7,8,9], at the hydroxyl group located at the C3 position of the A ring of the steroid nucleus. This modification reduces the availability of these steroids for CYP17A1-dependent metabolism and diverts them from downstream pathways leading to CYP21A2-dependent products (Figure 1). In addition, pregnenolone sulfate (P5-S) has been described as an important neurosteroid [10], indicating that SULT-dependent steroid metabolism may also contribute to the formation of compounds with intrinsic biological activity.

Previously, we showed that SULT1E1 and SULT2A1 can interact with CYP17A1, which led us to propose that these proteins may participate in a multienzyme complex mediating the sequential conversion of P5 to DHEA [11]. However, an additional level of regulation of steroid hormone biosynthesis may also be considered, involving interactions between SULTs and other key enzymes of this metabolic pathway. Biochemical evidence indicates that CYP enzymes can form functionally relevant complexes with non-redox protein partners. For example, the interaction of CYP26B1 with cellular retinoic acid-binding proteins CRABP-I and CRABP-II affects CYP26B1-mediated retinoic acid metabolism: holo-CRABPs can facilitate substrate delivery to CYP26B1, whereas apo-CRABPs inhibit its catalytic activity in a concentration-dependent manner [12]. More broadly, interactomic and SPR studies of other CYP-family enzymes, including CYP8A1 and CYP5A1, have revealed associations with multiple protein partners not involved in electron transfer, including glutathione S-transferases, molecular chaperones, and proteins involved in transport and cellular metabolism [13,14]. In addition, CYP17A1 and CYP21A2 have previously been shown to form protein–protein complexes [15], which further supports the possibility that steroid hormone biosynthesis may be regulated at the level of multienzyme complexes.

The aim of this study was to verify the hypothesis that CYP21A2 and SULT enzymes, which are involved in the regulation of steroid hormone biosynthesis, can interact with each other. Such interactions may modulate enzymatic activity or alter the availability of CYP21A2 substrates through their conversion into sulfated forms (Figure 1). Such enzyme–enzyme interactions could potentially facilitate enzyme colocalization and thereby contribute to the regulation of steroid metabolism. To address this hypothesis, we used surface plasmon resonance (SPR) biosensing, enzymatic activity assays, and molecular modeling. These data may provide insight into the role of SULT enzymes in the regulation of steroid hormone biosynthesis, where they may contribute to the fine-tuning of different enzymatic systems.

## 2. Results

### 2.1. SPR Analysis of CYP–SULT Interactions

**Figure 2:**
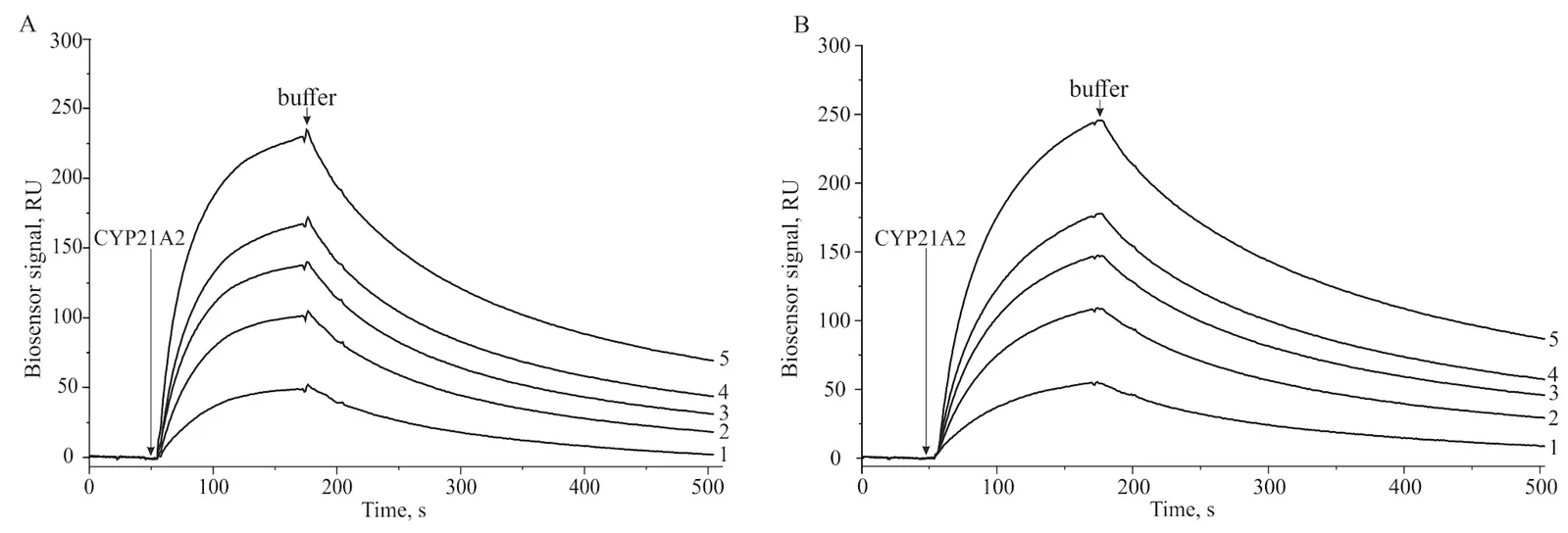
Sensorgrams showing the binding of CYP21A2 at different concentrations, 25 (1), 50 (2), 75 (3), 100 (4), and 150 nM (5), to immobilized SULT2A1 (**A**) and SULT1E1 (**B**).

SPR analysis was used to test the hypothesis that the studied SULT enzymes can interact with CYP21A2. Direct binding of purified recombinant CYP21A2 to immobilized SULT1E1 and SULT2A1 was detected in vitro by SPR. Representative sensorgrams of the interactions between the studied enzymes are shown in Figure 2. The Kd values of the studied protein–protein complexes were in the nanomolar range (Table 1). 3′-phosphoadenosine 5′-phosphosulfate (PAPS) at a concentration of 1 mM was found not to modulate these interactions.

To gain at least partial insight into the physicochemical basis of the interaction between the studied SULT enzymes and microsomal CYPs, we evaluated the thermodynamic parameters of the corresponding protein–protein interactions. To assess the thermodynamic heterogeneity of CYP–SULT complexes, we compared the thermodynamic parameters of CYP21A2–SULT1E1 and CYP17A1–SULT1E1 complexes, the latter having been previously described [11]. The SULT1E1–CYP21A2 and SULT1E1–CYP17A1 complexes differed in the thermodynamics of their formation (Table 2). The CYP21A2–SULT1E1 complex can be characterized as entropy-driven (−TΔS < 0), whereas the CYP17A1–SULT1E1 complex can be described as enthalpy-driven (ΔH < 0).

However, it should be noted that the number of experimental data points for which the temperature dependence of Kd was determined is limited (10, 20, 25, and 35 °C). Furthermore, the data obtained for the CYP17A1–SULT1E1 pair at 35 °C had to be excluded from the calculations. This was because this data point deviated significantly from the linear trends, likely due to enzyme denaturation under these conditions. For this reason, the thermodynamic parameter values obtained in this study for the protein–protein complexes should be interpreted with caution.

### 2.2. Enzymatic Assays

To assess whether the presence of CYP21A2 or SULTs affects each other’s enzymatic activities, in vitro activity assays were performed using the purified recombinant proteins. CYP21A2 was found to have no effect on DHEA sulfation by SULT1E1 or SULT2A1 (Supplementary Table S1). However, CYP21A2 exerted a moderate stimulatory effect on P5 sulfation by the studied SULT enzymes. In the case of SULT2A1, sulfation activity doubled, while for SULT1E1, it increased two-and-a-half-fold (Table 3). A moderate inhibitory effect of SULT2A1 on CYP21A2 activity was also observed (Table 3), whereas SULT1E1 did not affect the activity of the studied cytochrome P450. The addition of PAPS did not modulate the effect of SULTs on CYP21A2 activity, which is consistent with the SPR data.

### 2.3. Co-Expression Analysis

Our working hypothesis is that the steroidogenic enzymes considered in this study, namely SULT2A1/SULT1E1, CYP21A2, and CYP17A1, may exist in vivo as part of a multienzyme complex that contributes to the regulation of their function. In addition, we included another key enzyme of steroid hormone biosynthesis, 3β-HSD, in the analysis to evaluate its possible involvement in the regulatory processes considered here. Previously, we assessed the co-expression of CYP17A1 and CYP21A2 with SULT2A1/SULT1E1 and identified positive correlations between the expression of genes encoding these enzymes in several tissues and tumors, including pheochromocytoma/paraganglioma, minor salivary glands, certain regions of the nervous system, ovaries, and testes [11].

Therefore, in the present study, we analyzed co-expression for the following gene pairs: CYP21A2-CYP17A1, CYP17A1-HSD3B1, CYP17A1-HSD3B2, CYP21A2-HSD3B1, and CYP21A2-HSD3B2. The expression of HSD3B1 and CYP21A2 in the small intestine was negatively correlated, with R = −0.68. In contrast, the expression of HSD3B2–CYP17A1 and HSD3B2–CYP21A2 in pheochromocytoma/paraganglioma (PCPG) showed positive correlations, with R = 0.85 and R = 0.78, respectively. No significant co-expression correlations were detected for the other gene pairs of interest across the available list of tissues and tumors. The co-expression of CYP21A2-SULT was analyzed and described in our previous work [11].

### 2.4. In Silico Modeling of Protein Complexes

Since SPR analysis demonstrated direct binding between purified CYP21A2 and SULT2A1/SULT1E1 in vitro, we next explored possible structural arrangements of the corresponding binary complexes. AlphaFold2 MMseqs2 was used in silico modeling of two complexes: CYP21A2–SULT2A1 and CYP21A2–SULT1E1. In addition to AlphaFold2 MMseqs2 quality metrics, we used structural parameters obtained from PDBePISA model analysis.

Modeling was performed using both dimeric and monomeric forms of SULTs, because sufficient data on the functional activity of SULT oligomers in vivo are lacking [16]. For the CYP21A2–dimeric SULT2A1 complex, no models passing the quality criteria and visual inspection were obtained; therefore, this complex was excluded from further analysis. As a result, models of three CYP21A2 complexes with the studied SULT enzymes were obtained (Table 4; Supplementary Figure S1). Lists of interacting amino acid residues at the intermolecular interfaces of the modeled protein–protein complexes are provided in the Supplementary Tables S2–S4. However, AlphaFold2 models may not fully capture induced-fit dynamics during PAPS binding.

## 3. Discussion

Steroid hormone biosynthesis in humans involves numerous reactions and the enzymes that catalyze them. Protein–protein interactions are known to provide a basis for the colocalization of enzymes that catalyze functionally related, metabolically coupled steps of biochemical pathways [17,18]. This phenomenon may also be relevant to steroid hormone biosynthesis. We previously showed that human CYP17A1 interacts with SULT1E1 and SULT2A1 enzymes. In addition, CYB5A, an essential component of the enzymatic system responsible for DHEA synthesis, was found to interact with SULT2A1 (or SULT1E1) [11]. Based on these findings, we proposed that these protein–protein interactions may provide spatial colocalization of CYP17A1, CYB5A, and SULT2A1 or SULT1E1, thereby supporting the conversion of P5 into the sulfated form of DHEA.

The high physiological activity of steroid hormones, which regulate key functions in the body, requires strict control and multilevel regulation of their biosynthesis. One of the metabolic “switches” in steroid hormone biosynthesis may be the substituent at the C3 position of the A ring of the steroid nucleus (Figure 1). P5, which can be regarded as an entry point into the steroid hormone biosynthetic pathway, contains a 3β-hydroxyl group. The pathway then branches through the action of 3β-HSD, which catalyzes the formation of a keto group at C3 of the A ring of the steroid nucleus. Although this reaction is readily reversible, the presence of CYP21A2, which is characterized by unusually high processivity for a cytochrome P450 enzyme [19], promotes the active formation of glucocorticoid and mineralocorticoid precursors. In the absence of 3β-HSD activity, P5 can be converted by CYP17A1 into DHEA, a precursor of sex steroid hormones. However, human CYP17A1 catalyzes these reactions preferentially when a free 3β-hydroxyl group is present. Human cytosolic sulfotransferases, SULT2A1 and SULT1E1, can catalyze steroid sulfation by transferring a sulfo group to the 3β-hydroxyl group of the steroid nucleus (switch 1 on Figure 1). This modification strongly reduces their availability as substrates for 3β-HSD (switch 2 on Figure 1), CYP21A2, and CYP17A1. Therefore, sulfation may be considered a branch of steroidogenesis leading to the formation of mobile depot forms of steroids. Sulfated steroid forms can subsequently be returned to the steroidogenic pathway through the action of steroid sulfatase. Thus, cytosolic sulfotransferases may play a regulatory role in steroid hormone biosynthesis by diverting a fraction of steroids from the hydrophobic pool into soluble and mobile sulfated forms. This consideration led us to hypothesize that sulfotransferases may interact not only with CYP17A1 but also with CYP21A2. This assumption is further supported by previous evidence showing that CYP17A1 and CYP21A2 can interact with each other [15]. Such interactions may therefore have functional relevance to the regulation of steroid metabolism.

For this reason, it seemed reasonable to test the hypothesis that CYP21A2 can interact with SULT2A1 and SULT1E1. Using SPR biosensor analysis, we examined the ability of these enzymes to interact with each other. The SPR data confirmed the ability of these enzymes to interact with each other in vitro (Table 1, Figure 2). However, because these experiments were performed using purified recombinant proteins, they do not establish whether the same interactions occur in a physiological cellular environment. The CYP21A2 complexes with SULT1E1 and SULT2A1 showed similar kinetic parameters. As we previously reported, CYP17A1 complexes with SULTs form more slowly and dissociate more slowly than the CYP21A2 complexes with SULT1E1 and SULT2A1. This difference may reflect the fact that CYP17A1 and SULTs participate in a common biochemical pathway leading to DHEA synthesis, and such an enzymatic complex may therefore require a longer lifetime. By contrast, the interaction of SULT1E1 and SULT2A1 with CYP21A2 may be more closely related to the regulation of steroid biosynthesis. Judging by the affinity values, this interaction may also favor the preferential association of SULTs with CYP21A2 rather than with CYP17A1 under certain conditions.

Another notable observation is that PAPS modulated the formation of CYP17A1–SULT complexes in our previous study, whereas no such effect was observed for CYP21A2–SULT complexes. The mechanistic basis of this difference remains unclear. One possible explanation is that conformational or electrostatic changes associated with PAPS binding to SULTs affect the CYP17A1–SULT interface but do not substantially alter the CYP21A2–SULT interface. This suggests that the effect of PAPS may depend on the specific CYP partner and the structural organization of the corresponding protein–protein interface.

Since direct interaction between purified recombinant CYP21A2 and SULTs was detected in vitro, it was important to evaluate whether this interaction affects the enzymatic activity of the proteins involved. Such analysis may help clarify the functional significance of the complex. The activity assays revealed oppositely directed changes in the activities of the two enzymes participating in the complex, particularly for the CYP21A2–SULT2A1 pair (Table 3). Specifically, CYP21A2 increased SULT activity toward P5 sulfation, but not toward DHEA sulfation. These observations suggest that the presence of CYP21A2 may influence SULT-dependent P5 sulfation in vitro. However, since the activity assays were performed at a single CYP21A2:SULT molar ratio of 1:1, further concentration-dependent experiments are required to determine whether the observed effect is directly associated with the CYP21A2–SULT interaction. At the same time, SULT2A1 inhibited CYP21A2 activity (Table 3).

Thus, the regulatory role of SULT2A1 toward CYP21A2 may not be limited to the conversion of available potential precursors of 3β-HSD and CYP21A2 substrates into unavailable sulfated forms. In addition, the presence of SULT2A1 may modulate CYP21A2 enzymatic activity in vitro, though the underlying molecular mechanism remains to be established. A similar tendency was observed for the SULT1E1–CYP21A2 pair, although the effects were less pronounced. These findings are consistent with our previous data on the reciprocal effects of CYP17A1 with SULT2A1 and SULT1E1, where an increase in SULT activity in the presence of CYP17A1 was also observed [11].

At that time, we considered the CYP17A1–SULT interaction mainly in the context of DHEA conversion into its sulfated form and did not take into account the possibility that SULTs may also compete with CYP enzymes for upstream steroid substrates. In light of the present data, it is reasonable to assume that formation of the CYP17A1–SULT complex may have a dual functional role. On the one hand, it may facilitate the conversion of DHEA into DHEA sulfate. On the other hand, SULTs may divert pregnenolone into a sulfated form that is unavailable to CYP17A1. This may explain why SULTs did not directly inhibit CYP17A1-dependent DHEA formation in our previous study, while still potentially affecting substrate availability at the level of pregnenolone. Notably, SULT activity toward pregnenolone was not analyzed in our previous work. However, it is important to take into account the data on CYP17A1’s ability to hydroxylate P5-S to 17-OH P5-S in vitro in a reconstituted system and in the SOAT-HEK293 whole-cell system [20]. Remarkably, CYP17A1 in these conditions was not capable of catalyzing the 17,20-lyase reaction from 17-OH P5-2 to DHEA-S production. By the way, the absence of any CYP21A2 effect on DHEA sulfation in our work (DHEAS not being an enzymatic product within the sulfated branch) contrasts with the detectable reciprocal effects on pregnenolone sulfation (Table 3), consistent with pregnenolone/pregnenolone sulfate, rather than DHEA/DHEA-S, representing the potential principal node of the SULT2A1-CYP17A1.

Since we observed interactions of CYP21A2 with SULT2A1 and SULT1E1, the question arises as to the structure of these complexes. At present, no structural data are available for these complexes, as they were identified here for the first time. Therefore, to gain insight into the molecular basis of their formation, we performed molecular modeling, although this was not the main focus of the present study. Molecular modeling was carried out by predicting the three-dimensional structures of the complexes using AF2, followed by assessment based on internal quality metrics and subsequent visual inspection. The selected complexes were further evaluated using PDBePISA, which provided structural characteristics of the complexes, including interface area, the number of hydrogen bonds and salt bridges, and the estimated Gibbs free energy of complex formation (Table 4). PDBePISA was also used to obtain lists of amino acid residues forming the intermolecular interfaces (Supplementary Tables S2–S4). As a result, three models were selected: SULT1E1 dimer + CYP21A2, SULT1E1 monomer + CYP21A2, and SULT2A1 monomer + CYP21A2.

Given that SPR biosensor analysis provided thermodynamic data for the interactions between CYP21A2, CYP17A1, and SULT1E1 (Table 2), it was important to compare these data with the results of molecular modeling. Such comparison may help evaluate our hypotheses regarding the structural basis of the interactions. In the present study, as in our previous work, complexes involving SULT1E1 with CYP were modeled more reliably. Therefore, thermodynamic analysis of CYP–SULT interactions was performed using SULT1E1, CYP17A1, and CYP21A2.

In the model of the complex between dimeric SULT1E1 and CYP17A1, which corresponds to an enthalpy-driven interaction, 15 noncovalent interactions were predicted [11]. By contrast, the model of dimeric SULT1E1 with CYP21A2, which corresponds to an entropy-driven interaction, contained 7 such interactions (Supplementary Table S2). This observation is consistent with the thermodynamic characteristics of these complexes. No similar relationship was observed for monomeric SULT1E1: the complex with CYP17A1 formed 8 interactions [11], whereas the complex with CYP21A2 formed 8 interactions (Supplementary Table S3), despite the latter having a positive estimated ΔG value.

Comparative thermodynamic and structural analyses reveal distinct recognition mechanisms for the cytochrome P450 and sulfotransferase complexes. The CYP21A2–SULT1E1 complex is predominantly entropy-driven, exhibiting a positive enthalpy change and a significant entropy increase. This thermodynamic profile is typical for processes governed by the hydrophobic effect. The release of ordered water molecules from nonpolar surfaces provides a large positive entropy gain [21], which compensates for the unfavorable enthalpy required to break water–protein contacts. Such enthalpy–entropy compensation is a hallmark of hydrophobically mediated interactions [22]. Desolvation of nonpolar groups requires lower activation energy while yielding a greater entropy increase compared to polar contacts [23,24]. Accordingly, the density of hydrophobic residues (including proline, methionine, alanine, leucine, isoleucine, valine, phenylalanine, and tryptophan) at the interface of this entropy-driven complex is higher, averaging one hydrophobic residue per 48 Å^2^. In contrast, the CYP17A1–SULT1E1 complex is enthalpy-driven, characterized by a large negative enthalpy change and a substantial entropic penalty. The pronounced negative enthalpy (up to –174.2 kJ/mol) indicates a dense network of directional polar interactions, including hydrogen bonds and salt bridges that are significantly stronger at the low-dielectric interface than with the bulk solvent [25]. The corresponding unfavorable entropic contribution to the free energy (–TΔS of +142.0 kJ/mol) reflects the loss of conformational freedom due to the fixation of side chains and loops during the formation of a rigid complementary interface [26], alongside the loss of translational and rotational entropy upon association [27]. A lower density of hydrophobic residues, showing one hydrophobic residue per 63 Å^2^, supports this mechanism. Moreover, an analysis of the oligomeric state shows that models incorporating a dimeric form of SULT1E1 provide a much better fit to the experimental thermodynamic profile than models assuming the presence of monomeric SULT1E1 (for which the subunit interface parameters turned out to be virtually identical). Specifically, the number of noncovalent interactions per unit interface area in the complex of dimeric SULT1E1 with CYP17A1 was approximately 1.5-fold higher than that in the complex with CYP21A2, reinforcing the structural basis for the observed energetic differences. Nevertheless, it is important to bear in mind the limitations of our measurements of the thermodynamic parameters of protein–protein interactions in this study, as well as the need to exercise caution when interpreting the results of in silico structural modeling.

In our previous study, we showed that PAPS affected the protein–protein interaction of CYP17A1 with SULT1E1 and SULT2A1, which was consistent with molecular modeling results [11]. In the present study, however, PAPS did not affect the interaction of CYP21A2 with either SULT1E1 or SULT2A1. Therefore, we examined whether this observation was consistent with the molecular models of these complexes. But static models cannot fully reflect the PAPS-induced changes in the SULT structure.

The PAP-binding site was found to be located away from the intermolecular interface. However, part of the Tyr-231–Gly-252 loop of SULT2A1/PAP [28] or loop 3, comprising amino acids 234–262 in the SULT1E1 structure [29], was located within the contact region between the proteins. Crucially, previous structural and molecular dynamics studies have demonstrated that these specific loops are highly flexible and act as dynamic gates for cofactor and substrate binding. For example, the Tyr-231–Gly-252 loop in SULT2A1 transitions between open and closed conformations to regulate the active site [28], while loop 3 in SULT1E1 requires substantial pliability in the apo-state before transitioning into a rigid lid over the bound cofactor [29]. Thus, the interaction of CYP21A2 with either SULT1E1 or SULT2A1 may act to additionally secure the PAPS cofactor within its binding site by stabilizing this closed loop conformation.

Furthermore, this structural overlap suggests a dynamic functional coupling between the enzymatic cycle and complex dissociation. Upon completion of the sulfation reaction, the conversion of PAPS to the byproduct PAP requires the gating loop to reopen to allow for cofactor release. The subsequent expulsion of PAP and the mechanical opening of the loop could disrupt the protein–protein interface, thereby promoting the dissociation of the CYP21A2–SULT complex to facilitate cofactor exchange.

We therefore assume that the actual complex may differ to some extent from the AF2-predicted model. The discrepancy may partly stem from our simplified modeling. We examined only CYP21A2–SULT1E1 complexes, either dimeric or trimeric. One possible explanation is that, in a cellular environment, these proteins could participate in a larger multiprotein assembly whose organization differs from that predicted for isolated binary complexes. This possibility remains a working hypothesis and requires experimental validation. Such a complex could exhibit a different structural arrangement. This might allow the PAPS-associated loops to retain the mobility necessary for cofactor binding.

Molecular modeling was used to identify sites and amino acid residues that may be important for protein–protein interaction. The AlphaFold2 models showed that, for monomeric SULT1E1, the regions interacting with both CYP17A1 and CYP21A2 were represented mainly by individual amino acid residues. These included residues near the N-terminus of the sulfotransferase, namely Met-1 and Asp-6, followed by Asn-29, as well as a region near the C-terminus containing Lys-250 and Leu-251. None of these residues formed salt bridges or hydrogen bonds in both complexes; rather, they were common residues present within the intermolecular interface.

For dimeric SULT1E1, a more region-specific pattern of interaction with both studied cytochromes was predicted. Common interaction sites included residues Met-1 and Ser-3 at the N-terminus, the closely located pair Ala-146 and Asn-150 in the middle part of the chain, and regions containing Asp-244/Glu-245 and Lys-250/Leu-251/Ser-252/Pro-253 near the C-terminus. In both CYP17A1 and CYP21A2 complexes, dimeric SULT1E1 formed a salt bridge involving Met-1, whereas hydrogen bonds were formed via Met-1 and Lys-250. In the predicted tertiary structures, these interacting residue clusters were located close to each other (Supplementary Figure S2).

The structure of intraprotein channels was also analyzed using CAVER Analyst 2.0 for the SULT1E1dim–CYP21A2, SULT1E1mon–CYP21A2, and SULT2A1mon–CYP21A2 complexes. In all three modeled complexes, CAVER predicted potential channels connecting regions containing the active sites of SULT and CYP. In the models containing monomeric sulfotransferases, continuous putative channels extending between internal regions of the two proteins were identified. In the complex with dimeric SULT1E1, channels passing through the active sites of SULT and CYP and opening into the protein–protein interface region were observed (Supplementary Figure S3).

It should be emphasized that these intermolecular complexes were assessed only by molecular modeling, and further structural studies are required to refine these models. Nevertheless, the models provide possible structural arrangements for the experimentally observed binary interactions and are broadly consistent with the thermodynamic parameters determined by SPR.

## 4. Materials and Methods

### 4.1. Instrumentation

Protein–protein interaction analysis was performed using Biacore 3000 and Biacore 8K SPR biosensors (Cytiva, Marlborough, MA, USA).

Protein purification quality control was performed by MALDI-TOF mass spectrometry using a Microflex LRF instrument (Bruker Daltonik GmbH, Bremen, Germany). Mass spectra were processed using FlexAnalysis 3.0 software (Bruker Daltonics GmbH, Bremen, Germany) and were registered with the FlexControl 3.4 software (Bruker Daltonics GmbH, Germany).

HPLC analysis of enzymatic reaction products was performed using an Agilent Technologies 1200 Series chromatograph coupled to an Agilent 6410 Triple Quadrupole LC/MS system, and the results obtained were analyzed using MassHunter Workstation LC/MS Data Acquisition software for 6400 Series Triple Quadrupole v. 10.0 (Agilent Technologies, Santa Clara, CA, USA).

For LC-MS/MS protein identification in affinity purification–mass spectrometry (AP-MS) experiments, a Q Exactive HF-X mass spectrometer (Thermo Fisher Scientific, Waltham, MA, USA) was used.

### 4.2. Reagents

Progesterone (P4), pregnenolone (P5), NADPH, and 3′-phosphoadenosine-5′-phosphosulfate (PAPS) were obtained from Sigma-Aldrich (St. Louis, MO, USA).

HBS-EP+ buffer (150 mM NaCl, 3 mM EDTA, 0.05% detergent P20, 10 mM HEPES, pH 7.4), an amine coupling kit for covalent protein immobilization via primary amino groups, containing 1-ethyl-3-(3-dimethylaminopropyl)carbodiimide hydrochloride (EDC), N-hydroxysuccinimide (NHS), and 1 M ethanolamine hydrochloride (pH 8.5), as well as 10 mM acetate buffers (pH 5.0 and 5.5), were obtained from Cytiva (USA).

All other reagents were obtained from local suppliers.

### 4.3. Proteins

Highly purified recombinant full-length CYP21A2 [15], CYP17A1 [30], SULT2A1, and SULT1E1 [11] preparations, with purity exceeding 95% according to SDS-PAGE, were produced at the Institute of Bioorganic Chemistry of the National Academy of Sciences of Belarus. The proteins were obtained by molecular cloning and heterologous expression in a bacterial system (*E. coli*), followed by immobilized metal affinity chromatography via the hexahistidine tag, according to previously described protocols.

### 4.4. SPR Analysis of Kinetic and Equilibrium Parameters of Protein–Protein Complexes

SPR analysis of kinetic and equilibrium binding parameters was performed at 25 °C using a Biacore 3000 biosensor (Cytiva, USA). CM5 sensor chips coated with carboxymethylated dextran were used. The biosensor response was recorded in resonance units (RU), where 1 RU corresponds to the binding of approximately 1 pg of protein per mm^2^ on the sensor chip surface. Sensorgrams of protein–protein interactions were recorded as changes in the biosensor response, expressed in RU, over time.

SULT2A1 or SULT1E1 was immobilized by covalent coupling between carboxyl groups on the CM5 sensor chip surface and protein amino groups. The carboxyl groups were activated by injecting a freshly prepared 1:1 (*v*/*v*) mixture of 0.2 M EDC and 0.05 M NHS for 7 min at a flow rate of 5 µL/min. Proteins at a concentration of 15 µg/mL in 10 mM sodium acetate buffer were injected for 2 min at a flow rate of 5 µL/min. The buffer pH was 5.0 for SULT2A1 and 5.5 for SULT1E1. The chip surface was then washed with HBS-EP+ for 60 min.

CYP21A2 was injected as an analyte in HBS-EP+ at concentrations ranging from 50 to 150 nM through both the working channel and the reference channel without immobilized protein. The injection was performed for 2 min at a flow rate of 15 µL/min. Dissociation of the protein–protein complexes was monitored at the same flow rate for at least 5 min. The same experiment was performed in the presence of 1 mM PAPS to evaluate its modulatory effect on protein–protein interaction.

The resulting sensorgrams were obtained by subtracting the reference channel response from the working channel response and were analyzed using Biacore BIAevaluation software v. 4.1.1 with a 1:1 binding model including mass transfer, or using Biacore Insight software v. 3.0.11.15423 with the same model. The association rate constant (k*_on_*) and dissociation rate constant (k*_off_*) of the protein–protein complexes were determined. The resulting equilibrium dissociation constant (K_d_) is obtained as K_d_ = k*_off_*/k*_on_*.

### 4.5. Thermodynamic Parameters of Protein–Protein Interactions

A set of sensorgrams for the interaction of CYP21A2 and CYP17A1 with immobilized SULT1E1 was obtained at 10, 20, 25, and 35 °C using a multichannel Biacore 8K SPR biosensor (Cytiva, USA). CYP21A2 concentrations, flow rate, and contact time were the same as described above. CYP17A1 concentrations, flow rate, and contact time were the same as reported previously [11]. CYP17A1 was used at concentrations of 250, 500, 1000, and 2500 nM; the flow rate was 5 µL/min, and the contact time was 180 s. Temperature-dependent binding data used to calculate ΔG, ΔH, and −TΔS are presented in Table S5.

The Gibbs free energy change (ΔG) was calculated from Equation (1) using the obtained K_d_ values:

ΔG = R × T × ln K_d_, (1)

where T is the absolute temperature (K), and R is the universal gas constant (J × mol^−1^ × K^−1^).

The enthalpy change (ΔH) was determined from the dependence of lnKd on 1/T using a van’t Hoff plot, according to the linear form of the van’t Hoff equation (Equation (2)), as the slope of the plot with respect to the abscissa axis:

lnKd = (ΔH/R) × (1/T) − (ΔS/R).(2)

The entropic contribution (−TΔS) was calculated using Equation (3):

ΔG = ΔH − TΔS.(3)

### 4.6. CYP21A2 Enzymatic Activity in the Presence of SULTs

The enzymatic activity of CYP21A2 was assessed in the reduced system containing 1 μM of CYP21A2, 1 μM of CPR, and 50 μM of progesterone (P4) in a buffer comprising 25 mM of HEPES (pH 7.4) plus 10 mM of MgCl2. To estimate the effect of SULTs on CYP21A2 activity, 1 μM of SULT1E1 or SULT2A1 was added to the system (a molar ratio of CYP21A2:SULT was 1:1).

After incubating the protein aliquots at 37 °C for 10 min, the reaction was started by adding 250 µM NADPH. The reaction mixture was then incubated at 37 °C for 30 min. The reaction was stopped by adding 5 mL of methylene chloride, followed by centrifugation at 3000 rpm for 5 min. The upper organic layer was taken and evaporated in an argon atmosphere. After that, 100 µL of methanol was added to dissolve the precipitate.

Product formation was detected with HPLC using a C18 Luna column (100 Å, 250 × 4.6 mm) and an Agilent 1260 Infinity III (Agilent Technologies, Germany). Column elution was performed at a flow rate of 1 mL/min using a linear gradient from mobile phase A (62% methanol) to mobile phase B (100% methanol). The eluate was analyzed at a wavelength of 254 nm. The column temperature was 37 °C.

### 4.7. SULT Enzymatic Activity in the Presence of CYP21A2

SULT enzymatic activity was assessed in a reconstituted system containing 1 µM SULT and 25 µM PAPS in 50 mM Tris-HCl buffer (pH 7.5) supplemented with 10 mM MgCl2. To evaluate the effect of CYP21A2 on the catalytic activity of the studied SULTs, 1 µM CYP21A2 was added to the system at a CYP21A2:SULT molar ratio of 1:1.

Protein aliquots were preincubated at 37 °C for 10 min, after which the reaction was initiated by adding 50 µM DHEA or P5, followed by incubation at 37 °C for 30 min. The reaction was stopped by adding 5 mL of methylene chloride, followed by centrifugation at 3000 rpm for 5 min. The organic phase was collected and evaporated under argon. The residue was dissolved in 100 µL of acetonitrile.

The products of the enzymatic reaction of SULTs and DHEA-S were analyzed by LC-MS using a Zorbax Eclipse XDB-C18 column (80 Å, 150 × 4.6 mm, 5µm) and an Agilent Technologies 1200 LC–6410B Triple Quadrupole system (Santa Clara, CA, USA). Mobile phase A was 0.1 vol % formic acid in water, and mobile phase B was 0.1 vol % formic acid in a mixture of isopropanol–methanol (1:3). Isocratic elution was performed at 30% phase B for 1 min, followed by a linear gradient from 30 to 100% phase B over 1–10 min. The flow rate was 0.5 mL × min^−1^, and the column temperature was 40 °C. Injection volume—20 µL. Stop time—20 min. ASL temperature—+10 °C. Chromatographic separation was followed by APCI ionization in positive ion detection mode with the following parameters: gas temperature—+ 200 °C; gas flow drying gas—8 L/min; nebulizer—40 psi; capillary voltage—4000 V; corona current—4 µA; vaporizer—+ 400 °C; fragmentor voltage—80 V; mass range—100–1500 *m*/*z* in Scan mode.

For the quantitative assay, extracted ion chromatograms (EIC) for ions with *m*/*z* 271.2 for DHEA-S and DHEA were used. Solutions of DHEA-S and DHEA with concentrations of 100 and 200 µM in methanol were used as external standards.

Mass spectra of P5-S and P5 standards were also acquired in preliminary experiments. The main ion with *m*/*z* 299.2 for P5-S was assigned to [M + H − sulfate − H_2_O]^+^, whereas for P5 it corresponded to [M + H − H_2_O]^+^. The P5-S analysis was performed using the same method and the same equipment. Solutions of P5-S and P5 with concentrations of 100 and 200 µM in methanol were used as external standards.

MassHunter Workstation Software Qualitative Analysis v. B.07.00 (Agilent Technologies, Santa Clara, CA, USA) was used for processing spectra and chromatograms.

### 4.8. Method for Analyzing Co-Expression

Co-expression analysis of genes encoding the proteins of interest, including CYP21A2–CYP17A1, CYP17A1–HSD3B1, CYP17A1–HSD3B2, CYP21A2–HSD3B1, and CYP21A2–HSD3B2 pairs, was performed in normal tissues and malignant neoplasms using the GEPIA2 portal [31] (http://gepia2.cancer-pku.cn/#correlation, accessed on 14 December 2024). Pearson and Spearman correlation coefficients of R > 0.5, a number of clinical cases > 30, and *p* < 0.05 were used as cut-off criteria.

### 4.9. Structure Modeling of Protein Complexes

The possible structures of the studied protein complexes were predicted using the AlphaFold MMseqs2 pipeline [32] implemented on the ColabFold server v1.5.5. Default settings were used, except for the number of recycles, which was set to zero because recycles had a negligible effect on model quality in preliminary tests. Amino acid sequences of the canonical protein isoforms were obtained from the UniProt database [33].

Five models were generated for each complex. The following criteria were used to select working models: pLDDT (predicted local distance difference test) > 70 [34] and pTMscore (predicted template modeling score) > 0.5 [35]. The pLDDT score was used to assess the confidence of local protein structure prediction, whereas pTMscore was used to evaluate the predicted quality of the protein–protein interface.

Model visualization and energy minimization were performed in UCSF Chimera 1.17.3 [36]. We observed that some visually implausible models may still have acceptable quality metrics, including pLDDT and pTMscore. Therefore, the PDBePISA online resource [37] was used as an additional selection criterion. Only models with an interface contact area > 300 Å^2^ [38,39] and the largest number of interprotein salt bridges and hydrogen bonds were selected.

Prediction of channel structures within the protein–protein complex models was performed using CAVER Analyst 2.0 with a probe diameter of 0.9 Å [40].

## 5. Conclusions

In this study, we demonstrated direct binding between purified recombinant CYP21A2 and SULT1E1 or SULT2A1 in vitro using SPR. Enzymatic activity assays revealed increased P5 sulfation by both SULTs in the presence of CYP21A2 and decreased CYP21A2 activity in the presence of SULT2A1. These findings suggest a potential functional relationship between CYP21A2 and SULTs, although its underlying molecular mechanism and physiological significance remain to be established.

Molecular modeling provided possible structural arrangements for the experimentally observed binary interactions but also revealed certain limitations of the predicted structures, including possible restrictions on the mobility of SULT loops involved in PAPS binding. Additional co-expression analysis identified correlations involving *HSD3B2* and *CYP17A1*/*CYP21A2* co-expression in selected tissues. However, these correlations do not demonstrate physical association or complex formation. The possibility that CYP21A2, SULT1E1/SULT2A1, 3β-HSD, and other steroidogenic enzymes participate in a larger multiprotein assembly in cells therefore remains a working hypothesis requiring further experimental validation.

The chemical group at the C3 position of the A ring of the steroid nucleus may represent one of the molecular determinants influencing the direction of steroid metabolism. SULT1E1, SULT2A1, and 3β-HSD may contribute to the regulation of these metabolic branch points (Figure 1). Conversion of the 3β-hydroxyl group into a 3-keto group by 3β-HSD enables the formation of substrates for downstream CYP21A2-dependent pathways, whereas sulfation may redirect a fraction of steroid intermediates toward sulfated metabolic pathways. Thus, the proposed multiprotein assembly, if present in cells, may represent a highly organized enzymatic network with complex internal regulation.

## Figures and Tables

**Table 1:** Association rate constants (k*_on_*), dissociation rate constants (k*_off_*), and equilibrium dissociation constants (K_d_) characterizing the interaction of CYP21A2 with SULT2A1 and SULT1E1, as determined by SPR analysis. The interactions were detected at 25 °C in HBS-EP+ buffer.

Protein	k*_on_*, M^−1^s^−1^	k*_off_*, s^−1^	K_d_, M
Immobilized SULT2A1			
CYP21A2	(9.1 ± 0.2) × 10^5^	(8.6 ± 0.1) × 10^−3^	(9.5 ± 0.2) × 10^−9^
+PAPS	(9.6 ± 0.3) × 10^5^	(8.8 ± 0.2) × 10^−3^	(9.2 ± 0.4) × 10^−9^
Immobilized SULT1E1			
CYP21A2	(6.95 ± 0.13) × 10^5^	(7.98 ± 0.14) × 10^−3^	(1.15 ± 0.03) × 10^−8^
+PAPS	(7.3 ± 0.2) × 10^5^	(7.7 ± 0.2) × 10^−3^	(1.05 ± 0.04) × 10^−8^

**Table 2:** Thermodynamic parameters of CYP21A2–SULT1E1 and CYP17A1–SULT1E1 protein–protein complex formation, including Gibbs free energy change (ΔG), enthalpy change (ΔH), and the entropic contribution (−TΔS).

Complex	ΔG (kJ/mol)	ΔH (kJ/mol)	−TΔS (kJ/mol)
CYP21A2-SULT1E1	−41.6	31.2	−72.8
CYP17A1-SULT1E1	−32.2	−174.2	142.0

**Table 3:** Enzymatic activities of SULT2A1 and SULT1E1 (P5 sulfation) in the presence of CYP21A2, and activity of CYP21A2 (P4 hydroxylation) in the presence of SULT1E1 or SULT2A1 with or without PAPS. Control for SULT activities: without CYP21A2; Control for CYP21A2 activity: without SULTs and PAPS. PAPS was added at 25 µM. Data are means ± SD (n = 3) in min^−1^.

Additions	Enzymatic Activity, min^−1^
	SULT2A1
Control	0.37 ± 0.08
CYP21A2	0.67 ± 0.13
	SULT1E1
Control	0.03 ± 0.01
CYP21A2	0.08 ± 0.01
	CYP21A2
Control	0.88 ± 0.14
SULT1E1 + PAPS	0.76 ± 0.06
SULT1E1	0.72 ± 0.03
SULT2A1 + PAPS	0.41 ± 0.05
SULT2A1	0.47 ± 0.02

**Table 4:** AlphaFold2 quality metrics and structural characteristics of the intermolecular interfaces in CYP21A2 protein–protein complexes with SULT2A1 and SULT1E1.

Complexes	AlphaFold2		PDBePISA			
	pLDDT	pTM	Interface Area (Å^2^)	ΔG kcal/mol	H-Bonds (HB)	Salt Bridges (SB)
SULT1E1dimer + CYP21A2	83	0.5	963	−4.4	5	3
SULT1E1monomer + CYP21A2	85	0.6	1128	1.3	7	4
SULT2A1monomer + CYP21A2	83	0.6	1345	−2.6	12	10

## Data Availability

The raw data supporting the conclusions of this article will be made available by the authors on request.

## Supplementary Materials

The following supporting information can be downloaded at: https://www.mdpi.com/article/10.3390/ijms27219754/s1.

## Abbreviations

The following abbreviations are used in this manuscript:

3β-HSD	3β-hydroxysteroid dehydrogenase/Δ^5^–Δ^4^ isomerase
17OH P4	17α-hydroxyprogesterone
17OH P5	17α-hydroxypregnenolone
AF2	AlphaFold2
AP-MS	affinity purification–mass spectrometry
Chol	cholesterol
CPR	cytochrome P450 oxidoreductase
CYB5A	cytochrome b5 type A
CYP	cytochrome P450
CYP11A1	cholesterol side-chain cleavage enzyme
CYP11B1	steroid 11β-hydroxylase
CYP11B2	aldosterone synthase
CYP17A1	steroid 17α-hydroxylase/17,20-lyase
CYP21A2	steroid 21-hydroxylase
DHEA	dehydroepiandrosterone
DHEA-S	dehydroepiandrosterone sulfate
DOC	11-deoxycorticosterone
EDC	1-ethyl-3-(3-dimethylaminopropyl)carbodiimide hydrochloride
EDTA	ethylenediaminetetraacetic acid
HEPES	4-(2-hydroxyethyl)-1-piperazineethanesulfonic acid
HPLC	high-performance liquid chromatography
HSD3B1	hydroxy-delta-5-steroid dehydrogenase, 3 beta- and steroid delta-isomerase 1
HSD3B2	hydroxy-delta-5-steroid dehydrogenase, 3 beta- and steroid delta-isomerase 2
Kd	equilibrium dissociation constant
k*_off_*	dissociation rate constant
k*_on_*	association rate constant
LC-MS	liquid chromatography–mass spectrometry
MALDI-TOF	matrix-assisted laser desorption/ionization time-of-flight
NADPH	nicotinamide adenine dinucleotide phosphate
NHS	N-hydroxysuccinimide
P4	progesterone
P5	pregnenolone
P5-S	pregnenolone sulfate
PAP	3′-phosphoadenosine 5′-phosphate
PAPS	3′-phosphoadenosine-5′-phosphosulfate
PCPG	pheochromocytoma/paraganglioma
pLDDT	predicted local distance difference test
pTM	predicted template modeling score
RSS	Reichstein’s Substance S (11-deoxycortisol)
RU	resonance units
SDS-PAGE	sodium dodecyl sulfate–polyacrylamide gel electrophoresis
SPR	surface plasmon resonance
SULT	cytosolic sulfotransferase
SULT1E1	estrogen sulfotransferase
SULT2A1	dehydroepiandrosterone sulfotransferase
