# Supplementary material for: Fine-Tuning the Processes of Steroid Hormone Biosynthesis: SULT2A1/1E1 and CYP21A2 Interaction
Source: Int J Mol Sci. 2026 Oct 30;27(21):9754. doi: 10.3390/ijms27219754 (PMC13762974; doi:10.3390/ijms27219754)
Supplement: Supplementary file 1 [file ijms-27-09754-s001.zip › ijms-4618603-supplementary.pdf]

Fine-tuning the processes of steroid hormone biosynthesis: SULT2A1/1E1 and CYP21A2 interaction

Evgeniy Yablokov*, Anastasiya Tumilovich, Oksana Gnedenko, Diana Mednaia, Yuri Mezentsev, Pavel Ershov, Mihail Konstantinov, Leonid Kaluzhskiy, Tatsiana Tsybruk, Irina Grabovec, Polina Shabunya, Andrei Gilep, Alexis Ivanov

Correspondence: evgeyablokov1988@gmail.com

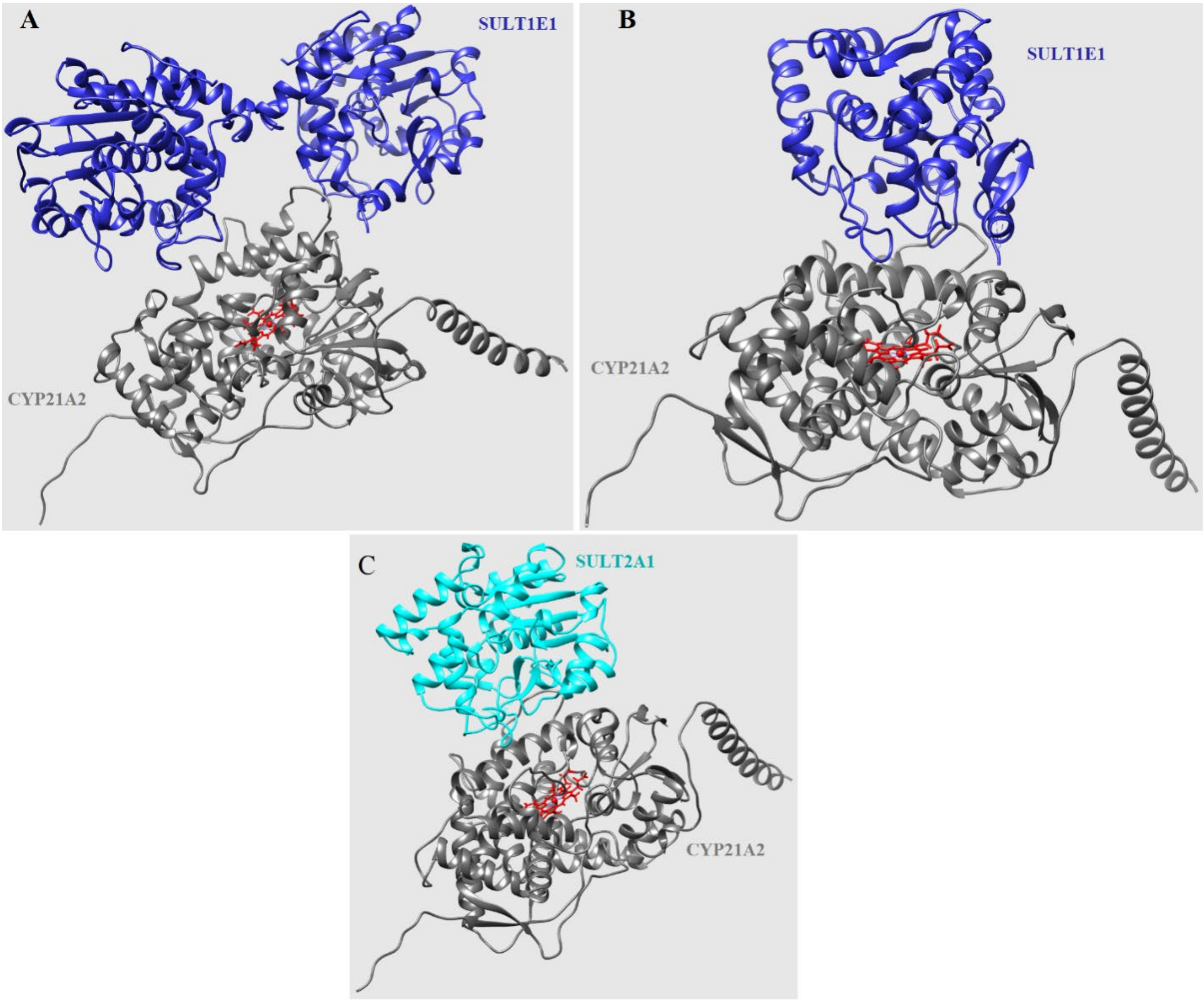

Figure S1. Models of dimeric (A), monomeric (B) SULT1E1, and monomeric SULT2A1 (C) with CYP21A2 predicted in the AlphaFold 2 software.

Table S1. Enzymatic activities of SULT2A1 and SULT1E1 (DHEA) in the presence of CYP21A2. Control for SULT activities: without CYP21A2.

**Additions**	**Enzymatic activity of SULT2A1, min^{-1}**
**Control**	0.02±0.00
**CYP21A2**	0.06±0.04
	**Enzymatic activity of SULT1E1, min^{-1}**
**Control**	0.11±0.03
**CYP21A2**	0.10±0.03

Table S2. A list of amino acid residues (a.a.r.) in the protein interfaces of complex of dimeric SULT1E1 with CYP21A2 (model).

**SULT1E1**			**CYP21A2**		
**a.a.r. position**	**a.a.r.**	**Bond**	**a.a.r. position**	**a.a.r.**	**Bond**
(1) 1	MET	SB	134	ASP	SB
(1) 6	ASP	SB	133	ARG	SB
(1) 245	GLU	HB	416	ASN	HB
(2) 1	MET	HB	370	ARG	HB
(2) 143	LEU	HB	272	GLU	HB
(2) 249	GLN	HB	276	GLN	HB
(2) 250	LYS	HB	277	LEU	HB
(1) 3	SER	-	338	LYS	-
(1) 9	GLU	-	381	GLU	-
(1) 150	ASN	-	414	GLY	-
(1) 248	ASN	-	418	ARG	-
(1) 250	LYS	-			
(2) 139	TYR	-	115	LEU	-
(2) 140	TYR	-	119	ALA	-
(2) 144	MET	-	265	VAL	-
(2) 145	VAL	-	266	ALA	-
(2) 146	ALA	-	267	GLN	-
(2) 148	HIS	-	268	PRO	-
(2) 149	PRO	-	269	SER	-
(2) 150	ASN	-	270	MET	-
(2) 151	PRO	-	271	GLU	-
(2) 244	ASP	-	273	GLY	
(2) 245	GLU	-	274	SER	-
(2) 248	ASN	-	275	GLY	-

(2) 251	LEU	-	278	LEU	-
(2) 252	SER	-	369	THR	-
(2) 253	PRO	-	371	PRO	-
(2) 256	ARG	-			
(2) 257	LYS	-			

(1) – first sulfotransferase monomer, (2) – second sulfotransferase monomer; SB – salt bridges, HB – hydrogen bonds; amino acids directly forming the indicated interactions with each other are highlighted in a separate box.

Table S3. A list of amino acid residues (a.a.r.) in the protein interfaces of complex of monomeric SULT1E1 with CYP21A2 (model).

SULT1E1			CYP21A2		
**a.a.r. position**	**a.a.r.**	**Bond**	**a.a.r. position**	**a.a.r.**	**Bond**
22	ASP	SB	118	LYS	SB
84	ARG	SB	271	GLU	SB
86	GLU	SB	118	LYS	SB
87	ASN	HB	115	LEU	HB
87	ASN	HB	122	LYS	HB
93	LYS	HB	269	SER	HB
240	THR	HB	133	ARG	HB
245	GLU	HB	125	ARG	HB
1	MET	-	76	ARG	-
2	ASN	-	80	GLU	-
5	LEU	-	83	VAL	-
6	ASP	-	84	LYS	-
9	GLU	-	85	LYS	-
10	LYS	-	86	TRP	-
25	LYS	-	113	TYR	-
26	TYR	-	119	ALA	-
29	ASN	-	126	SER	-
85	LYS	-	129	LEU	-
88	LEU	-	267	GLN	-
89	MET	-	268	PRO	-
90	ASN	-	270	MET	-
94	GLN	-	272	GLU	-
97	GLU	-	274	SER	-
146	ALA	-	337	TYR	-

235	PRO	-	338	LYS	-
241	THR	-	340	ARG	-
242	LEU	-	418	ARG	-
243	PRO	-	428	VAL	-
244	ASP	-	432	GLU	-
246	ILE	-	433	PRO	-
248	ASN	-			
250	LYS	-			
251	LEU	-			

SB – salt bridges, HB – hydrogen bonds; amino acids directly forming the indicated interactions with each other are highlighted in a separate box.

Table S4. A list of amino acid residues (a.a.r.) in the protein interfaces of complex of monomeric SULT2A1 with CYP21A2 (model).

SULT2A1			CYP21A2		
**a.a.r. position**	**a.a.r.**	**Bond**	**a.a.r. position**	**a.a.r.**	**Bond**
21	GLU	SB	84	LYS	SB
73	GLU	SB	118	LYS	SB
89	GLU	SB	370	ARG	SB
237	ASP	SB	125	ARG	SB
21	GLU	HB	84	LYS	HB
65	TRP	HB	272	GLU	HB
67	GLN	HB	272	GLU	HB
73	GLU	HB	118	LYS	HB
89	GLU	HB	370	ARG	HB
227	LYS	HB	264	GLY	HB
227	LYS	HB	122	LYS	HB
232	SER	HB	122	LYS	HB
232	SER	HB	126	SER	HB
237	ASP	HB	125	ARG	HB
15	THR	-	79	GLU	-
16	MET	-	83	VAL	-
19	ARG	-	85	LYS	-
22	THR	-	86	TRP	-
25	LYS	-	87	ALA	-
59	SER	-	88	ASP	-
62	ASP	-	113	TYR	-
64	LYS	-	115	LEU	-
68	SER	-	119	ALA	-
69	VAL	-	123	LEU	-

70	PRO	-	129	LEU	-
72	TRP	-	130	LEU	-
81	GLU	-	133	ARG	-
82	ILE	-	254	TRP	-
85	THR	-	260	TYR	-
86	ALA	-	267	GLN	-
90	THR	-	268	PRO	-
138	LYS	-	269	SER	-
222	SER	-	270	MET	-
225	GLU	-	271	GLU	-
226	ASN	-	273	GLY	-
228	MET	-	274	SER	-
233	LEU	-	277	LEU	-
234	LEU	-	371	PRO	-
235	SER	-	418	ARG	-
236	VAL	-	426	ALA	-
238	TYR	-	433	PRO	-
240	VAL	-			
241	ASP	-			
242	LYS	-			

SB – salt bridges, HB – hydrogen bonds; amino acids directly forming the indicated interactions with each other are highlighted in a separate box.

Table S5. Equilibrium dissociation constants (Kd) of complexes of CYP21A2 or CYP17A1 with SULT1E1 at different temperatures

T, °C / complex	K_d, M	
	CYP21A2–SULT1E1	CYP17A1–SULT1E1
10	1.19×10^{-7}	3.56×10^{-8}
20	4.53×10^{-8}	9.17×10^{-8}
25	5.16×10^{-8}	2.30×10^{-6}
35	3.83×10^{-8}	*2.79×10^{-8}

Notes. Data were obtained using a Biacore 8K in single-cycle mode. * - this point was excluded from the calculations.

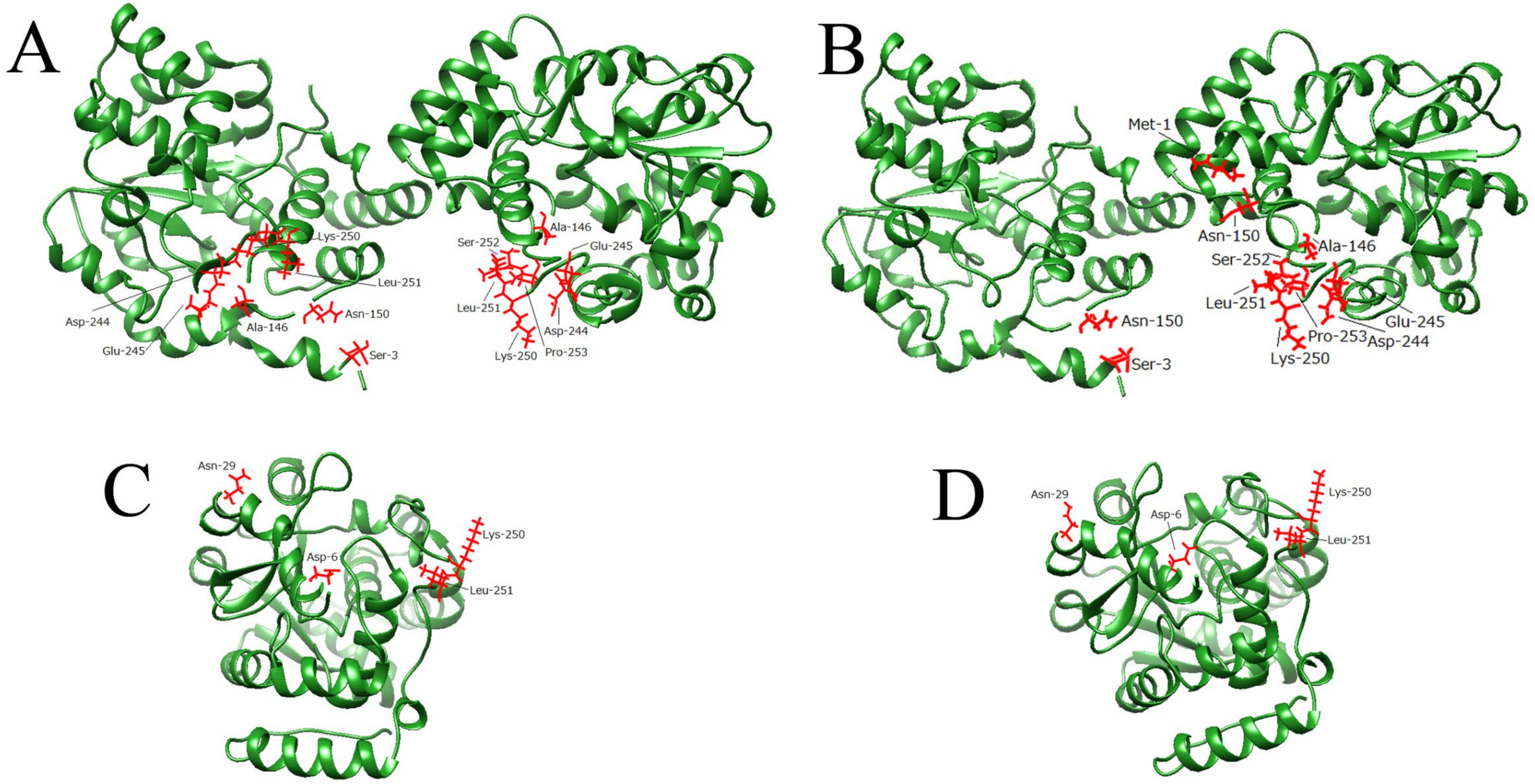

Figure S2. Amino acid residues of human SULT1E1 in dimeric (A, B) and monomeric (C, D) forms, forming contacts with CYP17A1 (A, C) and CYP21A2 (B, D).

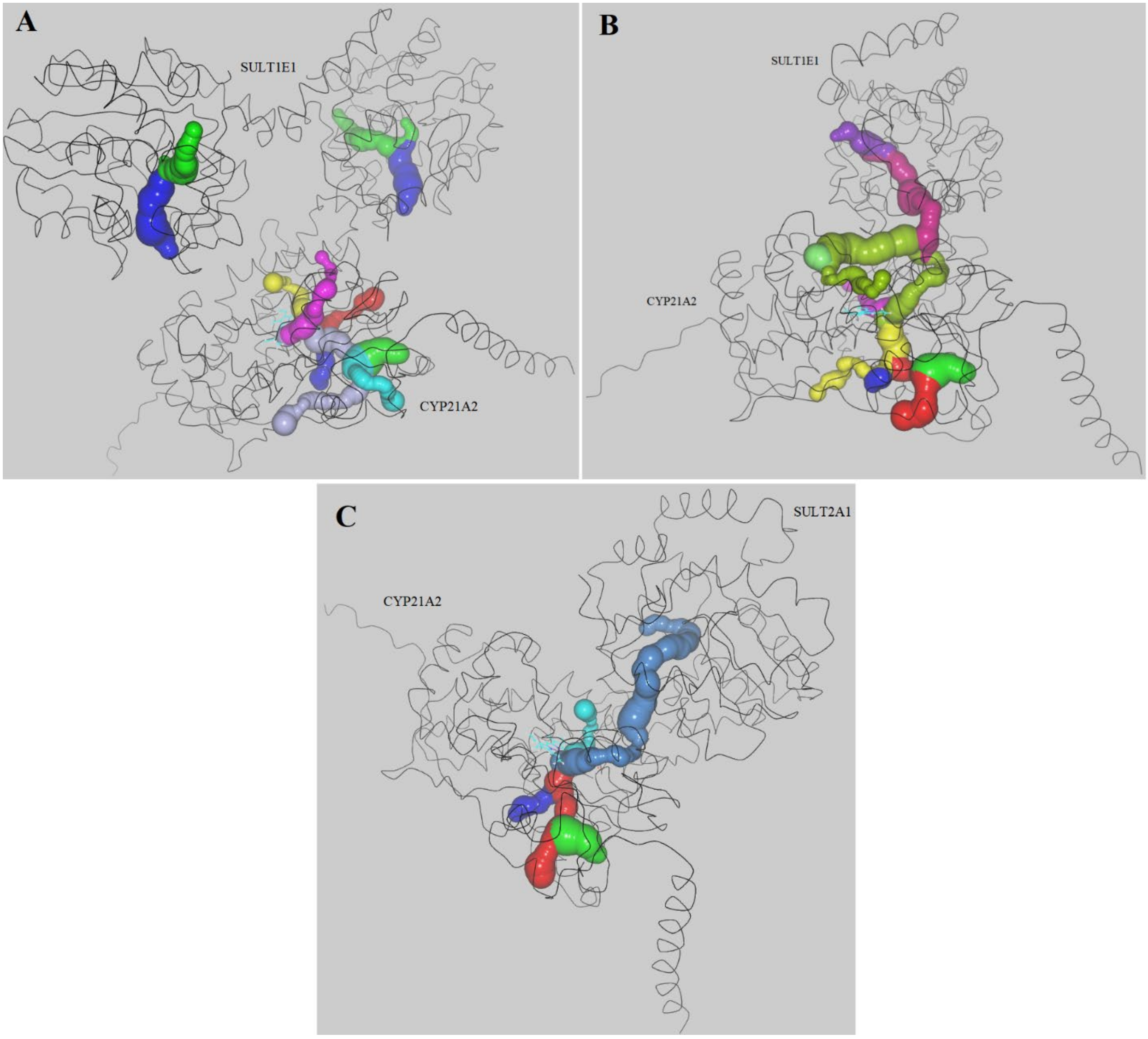

Figure S3. A set of calculated channels in CYP21A2 in complexes with the monomer (A) and dimer (B) forms of SULT1E1, and monomer form of SULT2A1 (C).
